# SALSA-Net: Explainable Deep Unrolling Networks for Compressed Sensing

**DOI:** 10.3390/s23115142

**Published:** 2023-05-28

**Authors:** Heping Song, Qifeng Ding, Jingyao Gong, Hongying Meng, Yuping Lai

**Affiliations:** 1 School of Computer Science and Communication Engineering, Jiangsu University, Zhenjiang 212013, China; songhp@ujs.edu.cn (H.S.); 2212008017@stmail.ujs.edu.cn (Q.D.); 2222208087@stmail.ujs.edu.cn (J.G.); 2 Jiangsu Engineering Research Center of Big Data Ubiquitous Perception and Intelligent Agriculture Applications, Zhenjiang 212013, China; 3 Electronic and Electrical Engineering Department, Brunel University London, Uxbridge UB8 3PH, UK; hongying.meng@brunel.ac.uk; 4 School of Cyberspace Security, Beijing University of Posts and Telecommunications, Beijing 100876, China

**Keywords:** compressed sensing, SALSA, deep unrolling, explainable networks, neural networks, image reconstruction

## Abstract

Deep unrolling networks (DUNs) have emerged as a promising approach for solving compressed sensing (CS) problems due to their superior explainability, speed, and performance compared to classical deep network models. However, the CS performance in terms of efficiency and accuracy remains a principal challenge for approaching further improvements. In this paper, we propose a novel deep unrolling model, SALSA-Net, to solve the image CS problem. The network architecture of SALSA-Net is inspired by unrolling and truncating the split augmented Lagrangian shrinkage algorithm (SALSA) which is used to solve sparsity-induced CS reconstruction problems. SALSA-Net inherits the interpretability of the SALSA algorithm while incorporating the learning ability and fast reconstruction speed of deep neural networks. By converting the SALSA algorithm into a deep network structure, SALSA-Net consists of a gradient update module, a threshold denoising module, and an auxiliary update module. All parameters, including the shrinkage thresholds and gradient steps, are optimized through end-to-end learning and are subject to forward constraints to ensure faster convergence. Furthermore, we introduce learned sampling to replace traditional sampling methods so that the sampling matrix can better preserve the feature information of the original signal and improve sampling efficiency. Experimental results demonstrate that SALSA-Net achieves significant reconstruction performance compared to state-of-the-art methods while inheriting the advantages of explainable recovery and high speed from the DUNs paradigm.

## 1. Introduction

Compressed sensing (CS) [1,2] theory exhibits a novel signal acquisition strategy where a signal can be recovered with overwhelming probability from far fewer acquired measurements than when resolved by the Nyquist sampling theory. This novel signal acquisition paradigm is much more hardware friendly and empowers image capturing with a sub-Nyquist sampling rate [3]. The core idea of CS is to sample and compress at the same time when the recovered signals have sparse representation in some transform domains. Applying CS theory to signal processing can reduce sampling time and cost while ensuring high signal reconstruction accuracy, which is of great significance for many practical applications such as remote sensing [4], single-pixel imaging [5], magnetic resonance imaging (MRI) [6], wireless sensor networks [7], radar imaging [8], spectral compressed imaging [9], computer vision, and pattern recognition [10].

The process of obtaining linear measurements in the theory of CS can be expressed as

(1) y=Ax,

where $y\in R^{m}$ is the compressed linear measurement, $x\in R^{n}$ is the original *k*-sparse (i.e., only *k* nonzero components in x) signal to be recovered, and $A\in R^{m\times n}$ is the sampling matrix. The CS sampling rate is defined as r=m/n. In this paper, we adopt a typical block-based image CS problem [11] which splits the high-dimensional natural image into non-overlapped B×B blocks and obtains linear measurements block by block with a relative smaller fixed sampling matrix for the subsequent reconstruction. Since m≪n, this inverse problem belongs to an underdetermined linear equation. When the sensing matrix A satisfies the constraint of isometry principle [12], the CS reconstruction problem is equivalent to

(2) $$\min_{x}{∥x∥}_{0},\;s.t.\;y=Ax.$$

The $l_{0}$-norm minimization problem is typically relaxed to the corresponding $l_{1}$-norm minimization problem, which is a convex optimization problem [13]:

(3) $$\min_{x}{∥x∥}_{1},\;s.t.\;y=Ax.$$

By appropriately choosing the regularization parameter λ>0, this problem can be further reformulated as an unconstrained optimization problem:

(4) $$\hat{x}=\arg\min_{x}\frac{1}{2}{∥y-Ax∥}_{2}^{2}+\lambda {∥x∥}_{1}.$$

The compressed sensing theory is dedicated to two main subproblems: signal acquisition and signal reconstruction. The signal acquisition problem focuses on designing efficient sampling matrices to reduce sampling complexity and sampling rate while ensuring reconstruction accuracy. The signal reconstruction problem is concerned with recovering the original signal from the undersampled measurements while satisfying fidelity and stability requirements. We divide existing CS signal reconstruction methods into traditional iterative solutions and deep neural network methods. In this section, we will briefly introduce both of them but focus on the network-based methods most relevant to our own.

### 1.1. Traditional Methods

In the past decades, various methods [13,14,15] have been developed to solve the problem (4), such as greedy-like algorithms [16], approximate message passing (AMP) [17], proximal gradient descent (PGD) [18], and alternating direction method of multipliers (ADMM) [19]. Greedy-like algorithms iteratively reconstruct a sequence of sparse signals based on support detection and signal recovering using truncated least squares optimization, such as Compressive Sampling Matching Pursuit (CoSaMP) [20], Subspace Pursuit (SP) [21], etc. The most representative PGD-based algorithms include the Iterative Soft Thresholding Algorithm (ISTA) [22], Fast Iterative Soft Thresholding Algorithm (FISTA) [23], and Non-Convex Evolutionary Sparse Target Algorithm (NESTA) [24]. ISTA is a simple iterative thresholding algorithm that uses prior information, such as the sparsity of a signal, to iteratively optimize and reconstruct the signal. FISTA improves upon ISTA by adding an acceleration term, resulting in better reconstruction quality in the same number of iterations. NESTA is a non-convex CS reconstruction algorithm that uses non-convex prior information and has shown good performance in high-dimensional sparse signal reconstruction. ADMM is an optimization method that solves sparse optimization problems by decomposing them into smaller subproblems each of which are then easier to solve. As an instance of ADMM, split augmented Lagrangian shrinkage algorithm (SALSA) [25,26] recasts the CS reconstruction problem into small-scale subproblems that are solved using soft thresholding operators. By iteratively solving the subproblems, SALSA converges significantly faster than ADMM. With well-studied signal formations, these approaches often take the advantage of strong convergence and theoretical analysis. However, they usually give rise to high computational complexity and suffer from choosing optimal prior and tuning parameters.

### 1.2. Network-Based Methods

Driven by the powerful learning ability of deep neural networks, deep-network-based compressed sensing reconstruction methods have been widely investigated. The core idea of these methods is to learn inverse mapping from compressed measurements simply to map a low-dimensional measurement vector to a high-dimensional image. These methods are usually divided into two categories: one is to train the reconstruction network as a black box classical deep model, and the other is an interpretable deep unrolling model [27] (algorithm unrolling [28] or model-based L2O [29]). In the first category of algorithms, Mousavi et al. [30] proposed a stacked denoising autoencoder (SDA) to solve statistical dependencies between signal elements. However, the fully connected network (FCN) used in SDA produces a large number of learnable parameters. Kulkarni et al. [31] proposed ReconNet, a deep learning model based on convolutional neural networks that captures local image information by directly learning the mapping relationship between compressed sensing measurements and image blocks and then assembling the reconstruction results of each block to achieve image reconstruction. In addition, ReconNet adopts the BM3D [32] algorithm as a denoiser to eliminate block artifacts in the output image. Yao et al. [33] proposed a method called DR${}^{2}$-Net, which improves ReconNet by adding residual learning to reconstructed images, achieving better results than ReconNet. Shi et al. [34] proposed a new algorithm called CSNet which learned the compressed sensing reconstruction process through convolutional neural networks while avoiding the problem of manually designing sampling matrices in traditional algorithms, reducing computation and improving reconstruction quality. However, this algorithm has a high-level requirement for training data and is weak in robustness to interference in the case of noisy data. Cui et al. [35] presented NLR-CSNet which aimed to learn a network that can reconstruct images from measurement vectors without pre-training and can achieve good results in low- and high-noise situations. Its non-local adaptive dictionary learning algorithm can learn more representative dictionaries, thus improving the robustness of the network. However, compared with other deep learning models, NLR-CSNet’s model is larger and requires a longer training time. Chen et al. [36] developed CASNet, which uses the adaptive sparse coding (ASC) method to obtain the sparse representation of input data and further improve the effect of sparse representation by using an adaptive threshold mechanism. Zhou et al. [37] proposed BCS-Net, which uses multiple channels to encode different frequency information of the image to improve reconstruction quality. You et al. [38] proposed COAST, which uses a multi-layer convolutional neural network (CNN) to reconstruct measurement values, including an encoder and a decoder. The encoder maps measurement values to a low-dimensional latent space, and the decoder reconstructs the information in the latent space into a complete image. However, the reconstruction speed of the COAST network is relatively slow and is not suitable for real-time applications. These deep models have the advantage of automatically learning features, avoiding the tedious process of manual feature design, and achieving a certain reconstruction effect. However, because they are black box models, they often cannot provide clear predictive explanations, making their use and debugging more difficult. At the same time, they are prone to overfitting when the dataset is small or the model is too complex, leading to training becoming more challenging.

The second type of deep unrolling model combines deep networks with iterative optimization algorithms and exhibits good interpretability. Gregor and LeCun [39] proposed the LISTA deep unrolling model in 2010, which adopts a multi-layer network structure to unroll the ISTA algorithm and uses neural networks to replace the threshold function in the ISTA algorithm for compressed sensing reconstruction of images. Following this seminal work, there has been a surge of efforts [40,41,42,43,44,45] that strive to propose deep unrolling networks by unfolding optimization-based algorithms. These deep unrolling models achieve explainable recovery and high accuracy, which have attracted increasing attention and have become the mainstream for image CS problems. However, these deep learning schemes adopt completely physics-free manners to directly unroll the optimization-based algorithm to learn recovery mapping from the measurements without explicitly making use of sampling processing and physical knowledge. To address these issues, physics-inspired methods incorporate both physical knowledge and sampling processing for further exploration. Zhang et al. [46] proposed the ISTA-Net model with trainable network modules to replace classic ISTA optimization and optimize all network modules through end-to-end learning. This introduced the independent learnable sparsifying/inverse transform with two convolutional layers separated by a Rectified Linear Unit (ReLU). Building on ISTA-Net, You et al. [47] proposed ISTA-Net++, which introduces feature enhancement modules to capture signal features and adds skip connection modules to accelerate network convergence. FISTA-Net [48] directly replaces the general nonorthogonal or even non-linear transform with four convolution layers separated by a ReLU, but no reasonable explanation is given. Yang et al. [49] presented ADMM-CSNet for CS-complex-valued MR imaging problems. The idea behind ADMM-CSNet is to replace the variable splitting and alternating optimization part in the ADMM algorithm with a deep neural network to fully utilize the non-linear mapping ability of neural networks. Liu et al. [50] proposed the RARE model, which uses unsupervised learning to obtain deep prior knowledge for image reconstruction. AMP-Net [51] trains the network using estimation errors generated during the iterative denoising process of the AMP algorithm, resulting in strong generalization performance. MAC-Net [52] introduces memory units in the network and uses double-threshold non-linear mapping and adaptive batch normalization to improve image quality and sparsity. Recently, more flexible backbone networks have emerged. CSformer [53] and TransCS [54] integrate Transformer self-attention-based hybrid architectures to obtain high-quality image recovery.

Interpretable deep unrolling models provide a way to solve image CS problems by incorporating physical knowledge into the model and making the training process more transparent. By absorbing the merits of both physics-free and physics-inspired image CS deep unrolling networks, we propose a novel optimization-based explainable deep unrolling network, coined SALSA-Net. The core idea of the SALSA-Net network is to truncate and unfold the iterations of the SALSA optimization algorithm and map or transform all the steps of each iteration into the end-to-end learning stage; then, all the stages will be concatenated to obtain a unified network. All the parameters involved in SALSA-Net, such as sparsifying/inverse transform, shrinkage threshold, and gradient steps are learned end-to-end. As a result, SALSA-Net takes the advantage of faster convergence and accurate recovery with well-defined explainability.

In summary, the main contributions of this paper are three-fold: (1) A novel deep unrolling model dubbed SALSA-Net is proposed for faster convergence of sparse reconstruction of image CS by mapping the updated steps of SALSA to deep networks. (2) Different from the traditional SALSA algorithm that requires manual tuning of gradient step size and regularization parameters, SALSA-Net learns all the parameters and applies physics-inspired constraints to ensure faster convergence. Furthermore, the sparsifying/inverse transformation in the residual domain is adopted to further improve image reconstruction accuracy. (3) Experimental results show that the proposed method achieves favorable performance against the state-of-the-art approaches in terms of both quantitative measure and visual quality.

## 2. Method

### 2.1. SALSA

This section examines the traditional reconstruction algorithm SALSA, which serves as the foundation for SALSA-Net. SALSA is an iterative algorithm designed to solve optimization problems involving both smooth and non-smooth convex functions. The algorithm’s fundamental concept is to divide the objective function into two components: a smooth portion and a non-smooth portion. During the iterative process, the smooth and non-smooth portions are updated separately using gradient descent or conjugate gradient methods, while the Lagrangian multiplier is updated using the augmented Lagrangian multiplier method. More specifically, to solve the problem (4), SALSA recasts it as the following two subproblems:

(5) $$\begin{array}{cc} \hat{x} & =\arg\min_{x}{∥y-Ax∥}_{2}^{2}+\mu {∥x-v+m∥}_{2}^{2}, \end{array}$$

(6) $$\begin{array}{cc} \hat{v} & =\arg\min_{v}\frac{\mu }{2}{∥x-v+m∥}_{2}^{2}+\beta {∥x∥}_{1}, \end{array}$$

where v is a vector with the same dimension as x, m is the Augmented Lagrange multiplier, and μ is a non-negative parameter used to control the weight of the Lagrange term. β is a non-negative parameter used to control the constraint of the L1 norm. By alternately solving the above two subproblems and updating the Lagrange multiplier m, the reconstruction result of the sparse signal can be iteratively obtained. Specifically, each iteration can be performed according to the following steps:

1. Augmented Lagrangian term minimization:
  (7) $$\hat{v}=\mathrm{Soft}\left(x+m;\;\beta /\mu \right),$$
  where Soft is a threshold function used to shrink the value of x to a nonzero value or zero. Its definition is
  (8) Soft(x,t)=sign(x)∗max(|x|−t,0).

2. Data term minimization:
  (9) $$\hat{x}={\left(\mu I+A^{T}A\right)}^{-1}\left(A^{T}y+\mu \left(v-m\right)\right),$$
  where **I** is the identity matrix.

3. Updated the Lagrange multiplier:
  (10) $$\hat{m}=m-v+x,$$
  where x are the results obtained by data term minimization.

Since SALSA decomposes problems into subproblems that handle only a part of the data at each iteration, it effectively handles large-scale problems. Moreover, SALSA converges faster than other classical algorithms such as ISTA and FISTA [25,26]. The SALSA algorithm has been widely applied in various fields, including image processing, computer vision, and signal processing.

### 2.2. SALSA-Net

**Figure 1:**
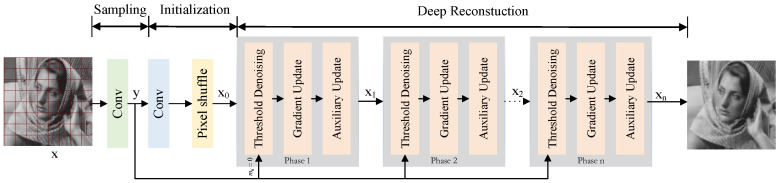
Illustration of SALSA-Net framework.

The traditional optimization model SALSA has high computational complexity, and current network models require improvement in restoring image details. To address these issues, this paper proposes a CS image reconstruction network based on a segmentation augmented Lagrangian algorithm. The network framework, shown in Figure 1, is divided into three parts: sampling, initialization, and deep reconstruction. The core algorithm used in this paper differs from ADMM-Net. Additionally, ADMM-Net uses random Gaussian matrix sampling, while SALSA-Net uses convolutional sampling. The structure of the reconstruction module also differs. ADMM-Net is divided into an encoder, decoder, and alternating direction multiplier network, while the reconstruction part of SALSA-Net is divided into a gradient update module, threshold denoising module, and auxiliary update module. We will provide a detailed description of the proposed model in the following subsections.

#### 2.2.1. Sampling

In block-based CS, the image is divided into non-overlapping blocks of size B×B×l, where B×B represents the spatial size of the image block and *l* represents the number of channels in the image. Each image block is considered an independent signal source, and a sampling matrix $A_{B}$ of size $N_{B}\times lB^{2}$ is utilized to measure the signals, where $N_{B}=rB^{2}$, *r* represents the sampling rate. Specifically, the sampling design of CSNet is followed, where each row of the sampling matrix *A* is treated as a convolution kernel, and a convolution layer is employed to simulate the block-based CS process. This convolution-based sampling method efficiently acquires CS measurements, avoiding the complexity and limitations of traditional hand-designed sampling matrices. The measurement results of each image block are represented as a feature map. This process can be represented using the convolution operator M(·)

(11) y=M(x).

The input image x is convolved with a non-overlapping convolution operation using a B×B×l-sized convolution kernel with a stride of B×B, which results in the output image y. In accordance with the block-based CS reconstruction method, *B* is set to 32. During training, the sampling network adaptively learns the sampling matrix, effectively utilizing the local structural features of the image to improve the accuracy and robustness of CS image reconstruction.

#### 2.2.2. Initial Reconstruction

**Figure 2:**
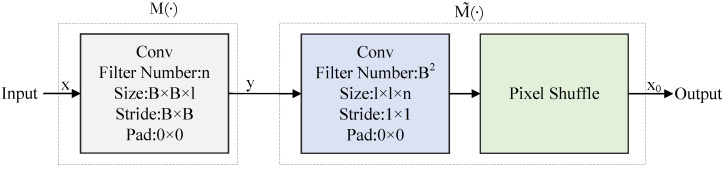
Illustration of the sampling and initial reconstruction process.

Block-based CS methods use the pseudo-inverse matrix of the sampling matrix to obtain the initial reconstruction of the image, denoted as $x=A_{B}^{†}\left(y\right)$ where $A_{B}^{†}$ is the size $1\times 1\times N_{B}$. In this paper, a convolution layer and a recombination concatenation layer are used to achieve the initial reconstruction, defined as follows:

(12) $$x_{0}=\tilde{M}\left(y\right),$$

where the CS measurements y serve as the input, which undergoes a convolution operation and pixel shuffle operation $\tilde{M}\left(\cdot \right)$ to obtain the initial reconstructed image $x_{0}$. Since the output of the sampling network is a $1\times 1\times N_{B}$ vector, the convolutional kernel size of the initial reconstruction layer is set to $1\times 1\times N_{B}$, with a stride of 1×1, to reconstruct each image block independently. Each image block is represented by a vector obtained through a convolutional layer and then recombined through concatenation to form the initial reconstruction image. The initial reconstruction network optimizes the entire reconstruction image, not just individual independent image blocks, thereby leveraging intra-block and inter-block information to better optimize the reconstruction. The sampling network and initial reconstruction network are depicted in the Figure 2.

#### 2.2.3. Deep Reconstruction

The core of the entire SALSA-Net is deep reconstruction, which is composed of multiple cascaded modules. Each module consists of a gradient update module (GUM), a thresholding denoising module (TDM), and an auxiliary update module (AUM).

**Figure 3:**
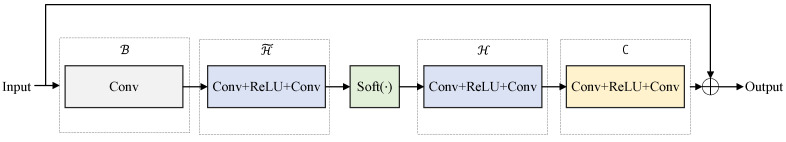
Illustration of the thresholding denoising module.

1. The thresholding denoising module v is designed to map the first iteration of the traditional SALSA algorithm onto the deep network architecture, aiming to eliminate the artifact noise in x+m using convolutional neural networks and the thresholding functions. The process can be expressed as:
  (13) $$\hat{v}=x+m+F\left(x+m\right),$$
  where F(·) is designed as a sequence of convolutional operations, which are specifically defined as:
  (14) $$F\left(\cdot \right)=B(\tilde{H}\left(L_{\mathrm{soft}}\left(H\left(C\left(\cdot \right)\right)\right)\right).$$
  The implementation of this module is illustrated in Figure 3, where C(·) is a one-shot convolutional operation that performs a linear transformation to increase the dimensionality of the input using 32 3×3 convolutional kernels; H(·) is designed to consist of two convolutional layers and a ReLU non-linear transformation layer to transform the output of C(·) into the desired domain and then perform denoising using a soft thresholding function $L_{\mathrm{soft}}\left(\cdot \right)$. The output is then transformed back to the original domain using the transformation $\tilde{H}\left(\cdot \right)$, satisfying $Hࢩ\tilde{H}=I$. Finally, a series of convolutional operations B(·) are used to achieve dimensionality reduction and obtain the final output *u*. The reconstructed results of C(·) and B(·) are stacked with the previous image residual information to obtain the updated reconstruction results. Unlike ISTA-Net, our B(·) is a deep network that learns the sparse representation of the input image using multiple convolutional layers and ReLU activation functions. Thus, B(·) becomes a trainable module that can adapt to different image scenes and tasks to improve the CS reconstruction performance.

2. The gradient update module is utilized in the SALSA algorithm to map the update process of x to the neural network. This module enables the learned M(·) from the sampling network to replace the sampling matrix A in the SALSA algorithm and the learned $\tilde{M}\left(\cdot \right)$ from the initial reconstruction network to replace $A^{T}$. This approach eliminates the need for manual design of the sampling matrix in traditional algorithms and allows for sharing of the convolutional kernel parameters with those of the sampling and initial reconstruction, thereby improving network performance. Moreover, the module utilizes the network training parameter step size μ to avoid manual parameter tuning. The process can be expressed as:
  (15) $$\hat{x}={\left(\mu I+M\left(\tilde{M}\left(x\right)\right)\right)}^{-1}\left(\tilde{M}\left(y\right)+\mu \left(v-m\right)\right).$$

3. The auxiliary update module is a linear combination of the previous two modules. Its main purpose is to accelerate the convergence speed of the algorithm, enabling a faster search for the optimal solution. Moreover, this module utilizes the computed m value as the initial value for the next iteration, which is integrated into the iterative computation. The process can be expressed as:
  (16) $$\hat{m}=m-v+x.$$

#### 2.2.4. Parameter and Loss Function Design

1. Parameters: The set of learnable parameters in our model comprises the transformation parameters M(·), $\tilde{M}\left(\cdot \right)$, C(·), H(·), $\tilde{H}\left(\cdot \right)$, and B(·). In addition, the step size μ and the shrinkage threshold β are also learnable, without the need for manual tuning. These parameters are shared across all steps of the reconstruction stage and are part of the deep neural network.
  To ensure the correct convergence of the parameters μ and β, we introduce some constraints in the following manner:
  (17) $$\begin{array}{c} \beta_{k}=\varphi \left(a_{1}k+b_{1}\right),\;a_{1}<0,\\ \mu_{k}=\varphi \left(a_{2}k+b_{2}\right),\;a_{2}<0. \end{array}$$
  Considering the decreasing noise variance during the iterative process, the shrinkage threshold is gradually decreased, and the step size should decrease smoothly during iterations. We enforce this constraint using the soft thresholding function φ(·)=ln(1+exp(·)). Since the network is fully shared and $\left\{a_{1},a_{2},b_{1},b_{2}\right\}$ is independent of iterations, we can use a different number of iterations for image reconstruction, as described in Section 3.

2. The design of the loss function: we define the original training set as ${\left\{x_{i}\right\}}_{i=1}^{K_{b}}$ and the recovered images as ${\left\{\tilde{x_{i}}\right\}}_{i=1}^{K_{p}}$, where $K_{b}$ is the number of images in the training set and $K_{p}$ is the total number of stages in the reconstruction network.

To facilitate comparative experiments, we quantify the difference between the original and reconstructed images using mean squared error. Inspired by sparse auto-encoders and block-based image reconstruction, we aim to minimize the difference between the reconstructed and original images. Therefore, we design the loss functions to include three parts:

(18) $$L_{\mathrm{total}}=L_{\mathrm{mse}}+\lambda_{1}L_{\mathrm{sym}}+\lambda_{2}L_{\mathrm{init}},$$

where

(19) $$\begin{array}{cc} L_{\mathrm{mse}} & =\frac{1}{K_{b}{K_{B}}^{2}}\sum_{i=1}^{K_{b}}{∥\tilde{x_{i}}-x_{i}∥}_{F}^{2}, \end{array}$$

(20) $$\begin{array}{cc} L_{\mathrm{sym}} & =\frac{1}{K_{u}}\sum_{i=1}^{K_{b}}\sum_{i=1}^{K_{p}}{∥\tilde{H}\left(H\left(u_{i}\right)\right)-u_{i}∥}_{F}^{2}, \end{array}$$

(21) $$\begin{array}{cc} L_{\mathrm{init}} & =\frac{1}{K_{b}}\sum_{i=1}^{K_{p}}{∥\tilde{M}\left(M\left(x_{i}\right)\right)-x_{i}∥}_{F}^{2}. \end{array}$$

The loss function of SALSA-Net consists of three parts: $L_{\mathrm{mse}}$ aims to minimize the difference between the original and reconstructed images, where ${∥.∥}_{F}^{2}$ represents the Frobenius norm of a matrix or tensor. $L_{\mathrm{sym}}$ aims to ensure symmetry by making the inverse transform of $\tilde{H}\left(\cdot \right)$ as close as possible to H(·), where $u_{i}=C\left(x+m\right)$, $K_{u}$ is the number of elements in $u_{i}$, and $K_{B}$ is the size of the image block. $L_{\mathrm{init}}$ is the constraint imposed on the initial reconstruction. Regularization parameters $\lambda_{1}$ and $\lambda_{2}$ are set to 0.01 and 0.001 by default.

## 3. Results

### 3.1. Training Configuration

This study utilized the widely used Train91 dataset to train the models, extracting a total of 88,912 randomly cropped image patches of size 33 × 33 as the training set to ensure a fair comparison of experimental results. The performance of the proposed approach was assessed on three benchmark datasets: Set5 [55], Set11 [52], and BSD68 [56]. Set5 comprises 5 color images, Set11 comprises 11 grayscale images, and BSD68 comprises 68 natural color images.

This study trained the model using six different sampling rates, 10%, 20%, 25%, 30%, 40%, and 50%, and evaluated reconstruction quality using the peak signal-to-noise ratio (PSNR) metric. Higher PSNR values indicate better reconstructed image quality. All models were trained for 160 epochs with a batch size of 64, a learning rate of 0.0001, and an initial bias value of 0. The initial values of $\left\{a_{1},a_{2},b_{1},b_{2}\right\}$ are set to −0.4, −0.2, −2, and −1. The experiments were conducted on a platform equipped with a Quadro RTX 6000 GPU.

### 3.2. Analysis of Experimental Results

In this section, we compared our proposed method with several benchmark algorithms, including TVAL3, ReconNet, ISTA-Net+, AMP-Net, NL-CSNet, MAC-Net, and ISTA-Net++. TVAL3 is a model-based method, ReconNet is a classic deep network method, and ISTA-Net+, AMP-Net, NL-CSNet, MAC-Net, and ISTA-Net++ are all deep unrolling methods. We made uniform modifications to all methods to address the block-based image problem.

**Figure 4:**
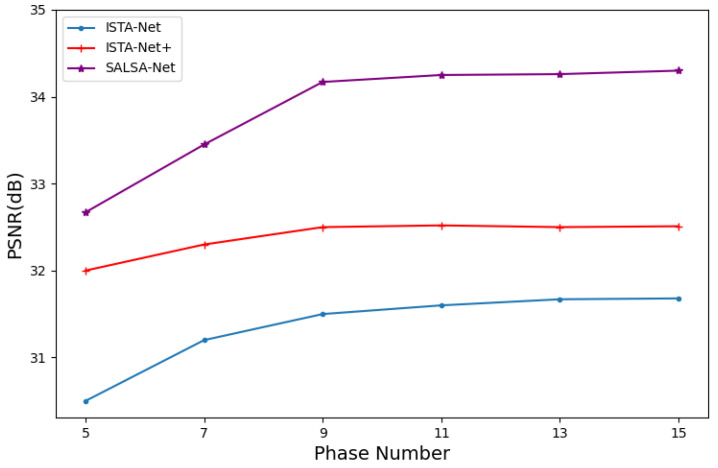
Comparison of average PSNR among ISTA-Net, ISTA-Net+, and SALSA-Net with different phases.

Firstly, to investigate the effect of the number of cascades in the network model on the reconstruction quality, we conducted a comparative analysis of our proposed method with ISTA-Net and ISTA-Net+ on the Set11 dataset with a 25% sampling rate. As depicted in Figure 4, the reconstruction quality of all methods improves as the number of cascades increases. Moreover, our proposed method outperforms ISTA-Net and ISTA-Net+ in terms of PSNR when the number of cascades is 7, 9, 11, 13, and 15. When the number of cascades exceeds 9, the improvement in reconstruction quality becomes insignificant. Therefore, to strike a balance between computational complexity and restoration performance, we set the number of cascades to 9 in the experiments conducted in this section.

**Figure 5:**
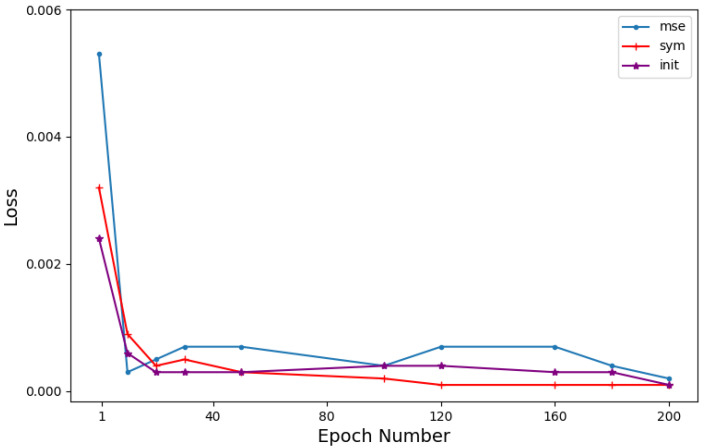
Updated diagrams of $L_{\mathrm{mse}}$, $L_{\mathrm{sym}}$, and $L_{\mathrm{init}}$ during the iteration process.

We conducted a performance comparison of three loss functions, namely, $L_{\mathrm{mse}}$, $L_{\mathrm{sym}}$, and $L_{\mathrm{init}}$, during the iterative process of SALSA-Net on the Set11 dataset with a sampling rate of 25%. Figure 5 illustrates that SALSA-Net demonstrates a consistent and rapid convergence towards zero on all three loss functions, indicating its excellent convergence properties.

**Figure 6:**
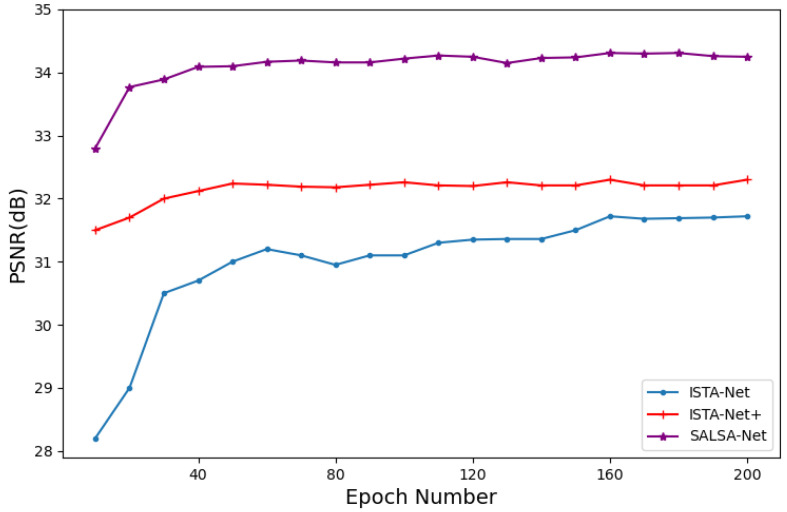
Comparisons of the average PSNR among ISTA-Net, ISTA-Net+, and SALSA-Net with different epochs.

To validate the convergence of our proposed method, experiments were conducted on the Set11 dataset with a sampling rate of 25% and N = 9. The proposed method was compared with ISTA-Net and ISTA-Net+ and the experimental results are presented in Figure 6. The experimental findings indicate that the proposed method outperforms ISTA-Net and ISTA-Net+ at different epochs. Notably, the proposed method exhibits superior performance after 40 epochs, while the three methods show a slow improvement in the subsequent epochs and slightly decrease after around 160 epochs. To balance computational complexity and reconstruction performance, we set the number of epochs to 160 in the subsequent experiments.

Table 1 presents the average PSNR of eight different algorithm models on the Set11 dataset under different sampling rates of 10%, 20%, 30%, 40%, and 50%. Bold numbers highlight the best reconstruction quality results at each sampling rate. The findings reveal that TVAL3 performs poorly at low sampling rates, while ReconNet exhibits poor performance at high sampling rates. The deep unrolling models (ISTA-Net+, AMP-Net+, NL-CSNet, MAC-Net, and ISTA-Net++) outperform classical compressive sensing models (TVAL3 and ReconNet) in terms of reconstruction results. Although the proposed SALSA-Net has slightly lower average PSNR than AMP-Net and ISTA-Net++ at sampling rates of 10% and 25%, respectively, it outperforms other reconstruction methods as the sampling rate increases, indicating its effectiveness in CS image reconstruction.

Table 2 and Table 3 present the comparison of the experimental results of SALSA-Net and other models on the BSD68 and Set5 datasets, respectively, to evaluate the generalization ability of SALSA-Net. The tables highlight the best results in bold. The findings of both tables indicate that SALSA-Net outperforms other algorithms at low sampling rates, exhibiting the highest average PSNR. Specifically, in BSD68, SALSA-Net achieves an average improvement of 0.67 dB over ISTA-Net++, 1.51 dB over MAC-Net, and 4.14 dB over ReconNet. In Set5, SALSA-Net achieves an average improvement of 0.13dB over AMP-Net, 0.67 dB over ISTA-Net++, and 6.37 dB over TVAL3. Moreover, SALSA-Net exhibits better performance than NL-CSNet, ISTA-Net+, and other algorithms at various sampling rates. These results demonstrate the good generalization ability and high performance levels of SALSA-Net.

**Figure 7:**
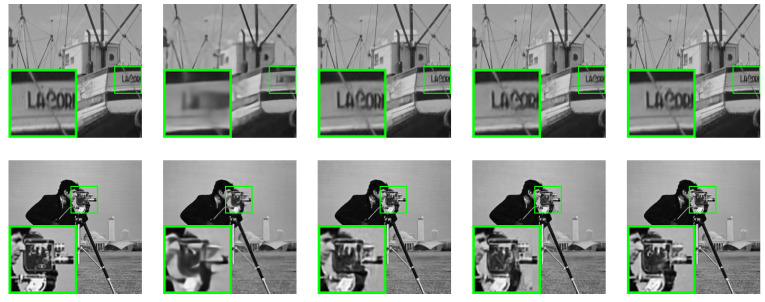
Visual comparisons of original image and reconstructions by ReconNet, ISTA-Net+, AMP-Net, and SALSA-Net on Set11 with a sampling rate of 25% after 160 epochs, arranged from left to right.

**Figure 8:**
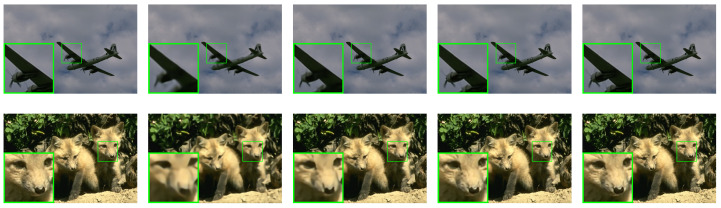
Visual comparisons of original image and reconstructions by ReconNet, ISTA-Net+, AMP-Net, and SALSA-Net on BSD68 with a sampling rate of 25% after 160 epochs, arranged from left to right.

**Figure 9:**
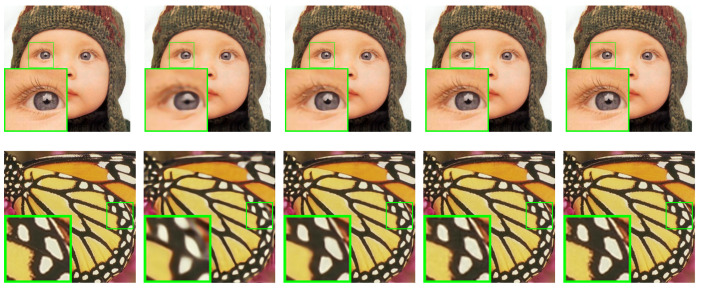
Visual comparisons of original image and reconstructions by ReconNet, ISTA-Net+, AMP-Net, and SALSA-Net on Set5 with a sampling rate of 25% after 160 epochs, arranged from left to right.

**Figure 10:**
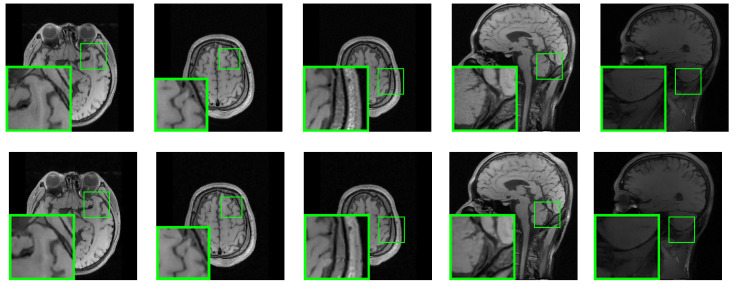
Visual comparisons of original brain images (**top**) and reconstructions by SALSA-Net (**bottom**) with a sampling rate of 25%.

This study conducted a comparative analysis of the proposed SALSA-Net method against four other methods using partial reconstruction images at a 25% sampling rate. The partial reconstruction results of Set11, BSD68, and Set5 are presented in Figure 7, Figure 8 and Figure 9, respectively. The reconstruction results of ReconNet were found to be blurry, while the other methods achieved effective reconstruction to a certain extent. Furthermore, to assess the scalability of SALSA-Net, a set of medical brain images was used to evaluate its performance on the CS-MRI reconstruction problem. As shown in Figure 10, the proposed method achieves CS-MRI reconstruction. The experimental results indicate that the SALSA-Net method can reconstruct texture structures clearly, exhibiting clear visual performance and signal reconstruction accuracy.

## 4. Conclusions and Future Work

In this paper, we proposed SALSA-Net, a deep unrolling network designed to address the compressed sensing problem with images. By combining the interpretability of SALSA with the powerful learning ability of deep networks, SALSA-Net incorporates learnable sampling and residual modules to achieve superior denoising and detail restoration performance. Extensive experiments on large datasets demonstrate the effectiveness of SALSA-Net’s sampling training and reconstruction strategy, which outperforms other state-of-the-art algorithms. In the future, we intend to introduce cross-area modules to further enhance the model’s performance and increase its flexibility. To expedite the study of deep unrolling networks, we will release the source codes and dataset of this paper to the public at https://github.com/songhp/SALSANet (accessed on 23 May 2023).

## Figures and Tables

**Table 1:** PSNR performance comparisons between SALSA-Net and seven other algorithms on Set11.

Methods	10%	20%	30%	40%	50%	Avg
**PSNR(dB)**						
TVAL3	22.99	27.92	29.23	31.46	33.55	29.03
ReconNet	24.11	25.52	28.71	30.77	31.59	28.14
ISTA-Net+	26.54	30.67	33.72	36.01	38.01	32.99
AMP-Net	**28.65**	31.05	32.91	35.31	37.45	33.07
NL-CSNet	28.11	31.43	33.82	35.60	37.13	33.22
MAC-Net	27.83	31.54	33.82	36.10	37.78	33.41
ISTA-Net++	28.34	**32.33**	34.86	36.51	38.73	34.15
SALSA-Net	28.49	32.25	**35.20**	**36.97**	**39.39**	**34.46**

**Table 2:** PSNR performance comparisons between SALSA-Net and seven other algorithms on BSD68.

Methods	10%	20%	30%	40%	50%	Avg
**PSNR(dB)**						
TVAL3	19.26	21.25	22.34	25.39	29.59	23.57
ReconNet	23.76	25.29	27.40	28.58	30.64	27.13
ISTA-Net+	25.29	27.23	30.03	32.23	33.56	29.67
AMP-Net	25.32	27.37	30.56	32.11	32.78	29.63
NL-CSNet	25.18	27.53	29.61	31.32	32.48	29.22
MAC-Net	25.34	28.43	30.11	31.37	33.58	29.76
ISTA-Net++	26.01	28.56	30.94	32.72	34.92	30.63
SALSA-Net	**26.96**	**29.12**	**31.55**	**31.55**	**35.60**	**31.27**

**Table 3:** PSNR performance comparisons between SALSA-Net and six other algorithms on Set5.

Methods	10%	20%	30%	40%	50%	Avg
**PSNR(dB)**						
TVAL3	27.12	30.35	32.49	34.77	36.63	32.27
ReconNet	26.99	29.34	31.44	33.62	35.41	31.36
ISTA-Net+	28.84	32.62	35.47	38.45	40.15	35.10
AMP-Net	33.31	**36.92**	39.14	41.12	42.07	38.51
MAC-Net	32.54	36.06	38.48	40.37	42.11	37.91
ISTA-Net++	33.04	36.41	38.86	39.92	41.64	37.97
SALSA-Net	**33.78**	36.63	**39.21**	**41.35**	**42.24**	**38.64**

## Data Availability

Not applicable.
